# Response of soil mites (Acari, Mesostigmata) to long-term Norway spruce plantation along a mountain stream

**DOI:** 10.1007/s10493-018-0314-3

**Published:** 2018-10-16

**Authors:** Jacek Kamczyc, Maciej Skorupski, Marcin K. Dyderski, Anna Gazda, Mariusz Hachułka, Paweł Horodecki, Izabela Kałucka, Marek Malicki, Remigiusz Pielech, Michał Smoczyk, Sylwia Wierzcholska, Andrzej M. Jagodziński

**Affiliations:** 1 Department of Game Management and Forest Protection, Faculty of Forestry, Poznań University of Life Sciences, Wojska Polskiego 71c, 60-625 Poznan, Poland; 2 Institute of Dendrology, Polish Academy of Sciences, Parkowa 5, 62-035 Kórnik, Poland; 3 Department of Forest Biodiversity, Institute of Forest Ecology and Silviculture, Faculty of Forestry, University of Agriculture in Kraków, 29 Listopada 46, 31-425 Kraków, Poland; 4 Department of Algology and Mycology, Faculty of Biology and Environmental Protection, University of Łódź, Banacha 12/16, 90-237 Lodz, Poland; 5 Institute of Forest Sciences, University of Łódź, Tomaszów Mazowiecki Branch, Konstytucji 3 Maja 65/67, 97-200 Tomaszów Mazowiecki, Poland; 6 Department of Botany, Institute of Environmental Biology, University of Wrocław, Kanonia 6/8, 50-328 Wrocław, Poland; 7 Rzepin, Poland; 8 Department of Botany, Institute of Biology, University of Opole, Oleska 22, 45-052 Opole, Poland; 9 Karkonosze National Park, Chałubińskiego 23, 58-570 Jelenia Góra, Poland

**Keywords:** River, Riparian forests, Understory biomass, *Picea abies*, Mite assemblages

## Abstract

During the nineteenth and twentieth centuries, coniferous monocultures were introduced, replacing natural broadleaved forests in Central Europe, mainly for economic benefits. In the mountains, Norway spruce [*Picea abies* (L.) H. Karst] was introduced in large areas previously covered with beech forests and also in natural riverside habitat corridors such as river valleys, despite its negative impact on the soil environment by e.g. organic matter accumulation, decrease of soil pH and changes in C/N ratio. We aimed to check how long-term Norway spruce plantations affect species richness and diversity of soil mites along a mountain river in former mixed and broadleaved forests. The study, based on 342 samples, was carried out in Stołowe Mountains National Park (SW Poland). Understory species biomass, soil pH and soil organic layer thickness significantly affected soil mite communities. Although coniferous forests did not differ from either broadleaved or mixed forests in mite density (number of individuals m^−2^) and species diversity (H′), they were characterized by low species richness and proportional abundance of Uropodina mites typical for broadleaved forests. In total, 4849 mites classified into 57 species were recorded from all forest types and no unique species were found in the sampled forests. Although the mite communities were dominated by the same common species (*Veigaia nemorensis*, *Paragamasus runcatellus*, *Leptogamasus obesus* and *Trachytes aegrota*), they still maintain the rare species of broadleaved forests and their high recovery potential may be used in forest conversion.

## Introduction

In recent centuries, almost all European forest ecosystems have been altered by forest management of varying intensities (Vanbergen et al. 2005). During the nineteenth and twentieth centuries, native broadleaved forests have been replaced by coniferous monocultures (mainly with *Pinus sylvestris* and *Picea abies*), which provided a higher economic return in both lowlands and highlands. However, these transformations have considerable environmental costs such as soil acidification, leaching of nutrients, frequent pest outbreaks and high windthrow susceptibility (e.g. Binkley and Valentine 1991; Binkley and Giardina 1998). Moreover, coniferous monocultures can affect forest biodiversity by their even-age tree structure and simple vertical arrangement (Zerbe and Wirth 2006). All those factors modify environmental conditions, i.e. light availability, temperature, moisture, litter and topsoil chemistry (e.g. Knight et al. 2008; Mueller et al. 2012) and therefore also determine microhabitat diversity (e.g. presence of coarse woody debris, tree hollows, veteran trees, plant patches, cavities, root plates, etc. which are pivotal for maintaining species diversity). These microhabitats are crucial for vascular plants (Zerbe et al. 2007), bryophytes (Jagodziński et al. 2018; Wierzcholska et al. 2018), fungi (Juutilainen et al. 2014), insects (Jukes et al. 2001) and birds (Smith et al. 2008). It should also be remembered that relatively homogeneous litter can create specific microhabitat conditions for different communities, as was proved for many groups of soil fauna including mites (Mueller et al. 2016). Moreover, in addition to the soil environment, some mite species are restricted in distribution to specific microsites, e.g. decayed wood (Laaksonen et al. 2008), tree hollows (Skubała and Gurgul 2011) or nests of birds (Napierała and Błoszyk 2013) or rodents (Krawczyk et al. 2015). Therefore, maintaining the diversity of microhabitats is crucial for species conservation and functional stability of forest ecosystems (Rusek 2001).

Streamside riparian forests are characterized by high microhabitat diversity and species richness (González et al. 2017). Riparian ecosystems play a role as ecological corridors and are the most diverse, dynamic and complex biophysical habitats in terrestrial ecosystems (Naiman et al. 1993). It is well known that riparian vegetation regulates light and temperature regimes (Naiman and Décamps 1990), which are crucial factors influencing detritus-based food webs (Seena et al. 2017). Moreover, riparian vegetation is one of the most threatened types of vegetation worldwide (Richardson et al. 2007). Therefore, it is very critical to understand how the longstanding, landscape-scale shifts from riparian broadleaved forests to coniferous monocultures affect soil ecosystems in the vicinity of streams.

We used soil mites (Acari, Mesostigmata) as bioindicators, because they are one of the most abundant groups of soil arthropods in temperate forests soils, occur with modest numbers of species and are very important for soil ecosystems, both in terms of species and function (Petersen and Luxton 1982). As Mesostigmata are the main predators among the soil mesofauna, by shaping communities of decomposers they are important regulators of decomposition processes and occupy a high trophic level in the soil decomposition food web (Schneider and Maraun 2009). Furthermore, they are highly susceptible to anthropogenic and natural disturbances and perturbations (Koehler 1991), which makes them good indicators of ecosystem processes (Gulvik 2007).

Several previous studies focused on Mesostigmata communities have been conducted in various types of forests (e.g. Diaz-Aguilar et al. 2013). Mites in Norway spruce forests have been studied in Central European mountains by few authors (e.g. Wierzbicka and Skorupski 2008). However, most of these studies did not include environmental data, such as soil pH, light availability, litter thickness, etc. or included it only partially (Manu 2011). Only a few studies were focused on mite communities along mountain streamsides or peat bogs in forests (Skorupski et al. 2008). It has been well proved that species richness of soil invertebrates depends on resource availability—soil and litter chemistry, light variability, as well as soil temperature (Mueller et al. 2016). Therefore, our study connects heterogeneity of soil microhabitats in coniferous monocultures along a mountain stream with the analysis of environmental data. This study also helps to understand the reaction of soil mesofauna to changes in forest soil environments after long-term coniferous plantation, which has been demonstrated in ‘common-garden’ experiments (Mueller et al. 2016).

We hypothesized that coniferous plantations affect diversity of mite communities in soil environments in streamside forests. Moreover, based on our previous study (Mueller et al. 2016) we assumed that availability of resources (light availability as a proxy for temperature and soil fertility as a proxy for prey abundance) will affect species composition of mite communities. The main goals of our study were: (1) to characterize the mite communities in soil environments along a stream, and (2) to check which environmental parameters are the main drivers of species composition of mite communities.

## Materials and methods

### Study site and design

The study was conducted in the Stołowe Mountains National Park, near the village of Karłów (50.47°N, 16.35°E), along the valley of the Czerwona Woda River in Stołowe Mountains, south-western Poland. The mountains represent a fragment of a great Cretaceous basin, which covers a wide region between the southern margin of Central Sudetes (SW Poland), Central Bohemia (Czechia) and Saxony (Germany) (Košťák 2001). These mountains are characterized by a mountain climate with mean annual temperature of 7.1 °C (1951–2000) in Kłodzko, located ca. 20 km away (Trouet and Van Oldenborgh 2013), and high precipitation in summer, with mean annual precipitation of 773 mm (1976–2000; Tarka et al. 2011). In the eighteenth and nineteenth centuries, the forests of the Stołowe Mountains, similar to other mountain areas in Europe, were transformed by human activity from natural broadleaved (with e.g. *Fagus sylvatica* L.) to coniferous monocultures, dominated by Norway spruce (*P. abies* (L.) H. Karst) (Wilczkiewicz 1982). Moreover, remnants of natural vegetation represented by riparian forests are represented by small patches and cover only a small part of riparian areas (Pielech 2015). Nowadays, admixture species on the territory of Stołowe Mountains National Park are represented by broadleaved *Acer pseudoplatanus* L., *Alnus incana* (L.) Moench, *Betula pendula* Roth., *Fagus sylvatica* L., *Sorbus aucuparia* L. and coniferous *Larix decidua* Mill. Moreover, stands within the National Park along the river valley are mature (80–120 years old). Therefore, the forests can be used to study the problem of how forest conversion impacts natural microhabitats in mountain broadleaved forests.

The Czerwona Woda River is the longest (13.5 km in length) river in Stołowe Mountains National Park. Elevations of its valley range from ca. 810 m near its spring to 600 m a.s.l. where it leaves the national park. The width of the river valley varies from 0.5 m near its spring to ca. 3 m at the end and therefore the total study area was restricted to 91.2 ha and a distance of ca. 9 km. Before sampling, we conducted a field inspection that revealed the occurrence of three main forest types along the river: (1) Norway spruce forests (mainly secondary forest community *Picea abies*–*Deschampsia flexuosa*, with patches of degraded *Vaccinio uliginosi*–*Piceeteum abietis* association (67.6 ha), (2) broadleaved forests—remnants of natural riparian forests from the *Alnion incanae* alliance (1.2 ha) and (3) mixed forests of *P. abies*, *A. pseudoplatanus*, *B. pendula*, *F. sylvatica* and *L. decidua* (ca. 2 ha). The remaining area was covered by non-forest ecosystems, mainly wet meadows (Pielech et al. 2018).

The study was carried out in all three forest types mentioned, using 171 study plots (25 m^2^; two samples per each plot). Most of the plots (160) were situated regularly every 100 m along the 9 km river segment, on both river banks. Additionally, 11 study plots were also established in sample plots of Pielech et al. (2018) to include vegetation survey plots. At each site, 2 subplots were established: the first subplot close to the river (1 m away from the watercourse) and the second in a different type of vegetation, at further distance (between 5 and 60 m away). Despite the low distances between the subplots in some cases, this approach provided different understory species composition, mainly driven by light regime. Among 171 established study plots, Norway spruce forests were represented by 162 plots, mixed forests by five plots and broadleaved forests by four plots, which reflects their cover in the area of the river valley studied (Pielech et al. 2018).

### Environmental factors measurements

For evaluation of impact of environmental factors on mite communities we used soil pH, light availability, soil organic layer thickness and understory vegetation parameters: biomass and main vegetation group cover. Soil organic layer thickness was measured at two points within each study plot, in each mite sample locality, and—in cases of big differences (over 100%) between two values—at a randomly selected third point. Light availability was expressed by DIFN (diffusive non-interceptance) measure of open sky fraction using an LAI-2200 device (Li-Cor Inc.). For each plot ten measurements in randomly selected points were taken. Soil pH was measured in solution in distilled water after 24 h of flotation using an electronic pH-meter. Understory biomass was determined for each plot within two circular samples (0.16 m^2^ each), from which aboveground parts of all understory vascular plants and mosses were harvested and dried to constant mass in an oven with forced air circulation at 65 °C (ULE 600, Memmert GmbH + Co. KG, Germany). Using data from the understory vegetation survey, for each plot we calculated cover proportion of two groups of plants species characteristic of the most abundant phytosociological classes (Mucina et al. 2016): *Carpino*-*Fagetea sylvaticae* and *Alno glutinosae*–*Populetea albae* (broadleaved and mixed forests) and *Vaccinio*-*Piceetea* (coniferous forests) (Table 1).

**Table 1:** Overview of study plot parameters and ranges

Variable	Mean ± SE	Median	Range (min–max)	1st quartile	3rd quartile
Distance from the river (m)	10.7 ± 0.7	5	1–60	1	15
DIFN (diffusive non-interceptance)	0.057 ± 0.004	0.035	0.003–0.751	0.017	0.066
Soil pH	4.02 ± 0.03	3.94	3.15–6.72	3.68	4.23
Soil organic layer thickness (cm)	4.4 ± 0.2	3.5	0.5–20	2	6
Understory biomass (g m^−2^)	91.43 ± 5.19	57.99	0.0–530.55	17.65	138.49
Cover proportion of *Carpino*-*Fagetea sylvaticae* and *Alno glutinosae*–*Populetea albae* classes plants in understory	0.046 ± 0.005	0.0	0.0–0.763	0.000	0.442
Cover proportion of *Vaccinio*-*Piceetea* class plants in understory	0.486 ± 0.016	0.47	0.0–0.965	0.251	0.761
Abundance per sample	14.2 ± 0.7	11	0–66	5	20
Species richness	4.9 ± 0.2	5	0–13	3	7
Shannon H′	1.180 ± 0.033	1.317	0.000–2.302	0.717	1.629
Density (no. individuals m^−2^)	3545.0 ± 167.2	2750	0–16,500	1250	5000

### Soil mite sampling

The soil sampling was conducted in late summer (September 2017) to coincide with high invertebrate abundance. Within each study plot two samples were taken with a steel corer ca. 40 cm^2^ and 10 cm deep. Generally, due to the sampling design, we have obtained three types of samples, i.e., coniferous monocultures with Norway spruce (n = 324), mixed (10) and broadleaved (8) which differed in numbers but represented their proportional abundances on the whole study area. Overall, 342 soil samples were taken and transported afterwards to the laboratory (Poznań University of Life Sciences, Poland) in plastic bags. Then, samples were extracted separately in Tullgren funnels for 7 days with 75% alcohol as a preservative. Mesostigmata mites were subsequently selected from the samples using a stereomicroscope at 10–25 × magnification, cleared in 85% lactic acid for a minimum of 5 days, depending on the degree of transparency required for each specimen, mounted on slides using Hoyer’s medium and finally dried at 45 °C for a minimum of 7 days, using a slide warmer. Then mites were identified to species (adults and juveniles when possible) or genus level using a light microscope and identification keys (e.g. Micherdziński 1969; Karg 1993).

### Data analysis

Each sample provided an independent estimate of local (point) diversity and abundance and finally produced one data point for the analyses of species composition and species accumulation. To assess species richness, diversity and abundance in forest types we used data pooled per study plot. Abundance data were recalculated into density (number of individuals m^−2^) for direct comparison with other published data. Diversity for each sample was measured using Shannon’s diversity index (*H*′). We have also analyzed differences in distributions of two taxonomic groups (Uropodina and Gamasina) among habitat types using Chi squared tests, implemented in the *stats::chisq.test()* function. We also prepared species accumulation curves to assess both sampling effort and the relationship between number of samples and overall species richness. Curves were calculated and plotted using the *vegan::specaccum()* function (Oksanen et al. 2018).

To assess the relationships between environmental parameters and mite communities we used the Redundancy Analysis (RDA) method of ordination, implemented in the *vegan::rda()* function (Oksanen et al. 2018). RDA is literally a method of multidimensionality reduction into linear combinations of input parameters (species abundances transformed using Hellinger’s square root transformation), which explains most of variation within parameters. Prior to analyses we excluded empty samples from analysis, thus final n = 323. RDA was chosen due to short (linear) gradient length. In preliminary analyses we compared RDA and canonical correspondence analysis (CCA) using amount of variance explained and proportion of variance explained by particular axes (using diagnostic scree plots). RDA, as a constrained analysis, reveals the similarity between samples along a priori assumed environmental variables. We assumed that mite species composition would depend on forest type and the environmental parameters studied. In the primary model we used all these variables and then we reduced them to obtain the model with the lowest Akaike Information Criterion (AIC) by forward selection of variables. To assess the statistical significance of the variables we used permutation tests for analysis of variance (PERMANOVA), implemented in the *vegan::anova.cca()* function (Oksanen et al. 2018). To compare differences of mite communities and environmental parameters among tree stand types we used Kruskal–Wallis tests (KW), due to lack of normality of distributions. To assess the differences between tree stand types assessed using Kruskal–Wallis tests we used Fisher’s *posteriori* test. This approach was implemented in the *agricolae::kruskal()* function. These analyses were performed using R software (R Core Team 2018).

## Results

### Abundance, species richness and community structure

**Fig. 1:**
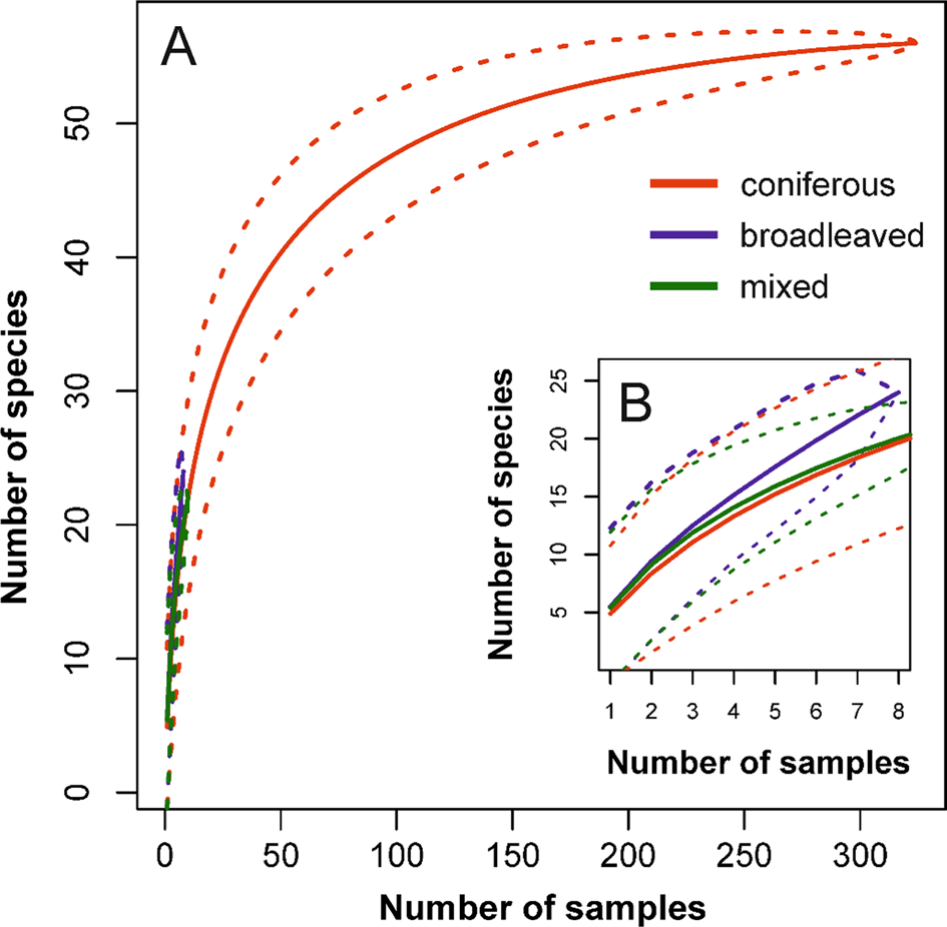
Species accumulation curves for the forest types studied (**a**) and magnification of the first part of the curves (**b**). Dashed lines indicate range of SE

In total, 4849 mites were collected and classified into 57 species. The mites represented two suborders: Gamasina (4144 individuals, 13 families) and Uropodina (705 individuals, 4 families). Overall, Parasitidae, with 14 species, was the most abundant family. Our study indicated that three microhabitats analyzed were inhabited by the same common species (Table 2). Total number of species recorded differed among forest types. The highest species richness was recorded from coniferous forest (57 species), and lower values from both mixed (25) and broadleaved (20) forests. Analysis of species accumulation curves (Fig. 1b) revealed similar patterns of species accumulation in each of the habitats studied in the parts of the curves with low numbers of samples. However, the end of the curve for broadleaved forests was directed more upwards than those of mixed and coniferous forests. In coniferous forests after ca. 150 samples the curve reached a plateau. Analysis of the proportional abundance of Gamasina and Uropodina revealed lack of statistically significant differences among forest types (KW: χ^2^ = 1.147, *df *= 2, *p* = 0.56). The percentage of Gamasina versus Uropodina changed from mixed (90.2% Gamasina vs 9.8% Uropodina) through broadleaved (87.2 vs 12.8%) to coniferous (85.3 vs 14.7%). The most abundant species in mite communities was *Veigaia nemorensis*, which dominated in all forests (32.6, 28.7 and 24.8% of individuals in broadleaved, coniferous and mixed forests, respectively). The next most abundant species within the study plots in all types of forests were *Paragamasus runcatellus* and *Leptogamasus obesus* which were common and usually dominate in coniferous forests in the Sudetes Mts. *Gamasellus montanus* and *Trachytes aegrota* were also dominants on plots in spruce stands, but these species as well as *Pachylaelaps bellicosus* were still dominants or even increased in abundance on plots in mixed and broadleaved forest. Moreover, we have recorded other species i.e. *Macrocheles* sp., *Olodiscus minima*, *O*. *misella*, *Pachylaelaps longisetis*, *Polyaspinus cylindricus*, *Trachytes montana*, *Uropoda cassidea*, *U. splendida* and *Veigaia mollis.*

**Table 2:** Abundance (no. individuals) of mites in three forest types along the Czerwona Woda River in Stołowe Mountains National Park

Species	Abundance (individuals)		
	Broadleaved	Mixed	Coniferous
	n = 8	n = 10	n = 324
*Amblyseius* sp.	0	0	4
*Dendrolaelaps* sp.	0	0	1
*Dermanyssus gallinae* (De Geer, 1778)	0	0	1
*Epicrius resinae* Karg, 1971	0	0	4
*Eviphis ostrinus* (C. L. Koch, 1836)	0	1	75
*Gamasellodes bicolor* (Berlese, 1918)	0	0	2
*Gamasellus montanus* (Willmann, 1936)	0	6	260
*Geholaspis longispinosus* (Kramer, 1876)	0	1	3
*Geholaspis mandibularis* (Berlese, 1904)	1	0	6
*Geholaspis pauperior* (Berlese, 1918)	9	11	37
*Holoparasitus tirolensis* (Sellnick, 1968)	1	1	70
*Hypoaspis* (*Pneumolaelaps*) *procera* Karg, 1965	0	0	15
*Hypoaspis aculeifer* (Canestrini, 1883)	3	2	86
*Hypoaspis praesternalis* Willmann, 1949	0	0	6
*Hypoaspis vacua* (Michael, 1891)	0	0	3
*Iphidosoma physogastris* Karg, 1971	0	0	5
*Lasioseius lawrencei* Evans, 1957	0	2	4
*Leioseius magnanalis* (Evans, 1958)	1	0	22
*Leptogamasus cristulifer* (Athias-Henriot, 1967)	0	1	8
*Leptogamasus obesus* (Holzmann, 1955)	1	26	343
*Leptogamasus parvulus* (Berlese, 1903)	0	1	4
*Leptogamasus suecicus* (Trägardh, 1936)	2	8	216
*Macrocheles* sp.	0	0	3
*Olodiscus minima* Kramer, 1882	0	0	13
*Olodiscus misella* (Berlese, 1916)	1	4	43
*Pachylaelaps bellicosus* Berlese, 1920	0	14	64
*Pachylaelaps furcifer* Oudemans, 1903	0	2	57
*Pachylaelaps longisetis* Halbert, 1915	7	3	14
*Paragamasus puerilis* (Karg, 1963)	4	8	600
*Paragamasus runcatellus* (Berlese, 1903)	0	0	54
*Paragamasus similis* (Willmann, 1953)	0	0	2
*Paragamasus vagabundus* (Karg, 1968)	0	2	96
Parasitidae (Unidentified juvenile)	0	0	10
*Parazercon radiatus* Berlese, 1910	0	1	32
*Pergamasus* (*Thenargamasus*) sp.	0	0	2
*Pergamasus barbarus* (Berlese, 1905)	0	0	2
*Pergamasus brevicornis* (Berlese, 1903)	0	0	11
*Pergamasus mediocris* Berlese, 1904	0	0	20
*Pergamasus* sp.	0	0	10
*Polyaspinus cylindricus* Berlese, 1916	0	1	30
*Porrhostaspis lunulata* Müller, 1859	0	1	8
*Prozercon kochi* Sellnick, 1943	0	2	30
*Rhodacarellus silesiacus* Willmann, 1935	0	0	11
*Trachytes aegrota* (C. L. Koch, 1841)	16	34	310
*Trachytes montana* Willmann, 1953	0	4	67
*Trachytes pauperior* Berlese, 1914	0	3	95
*Urodiaspis tecta* (Kramer, 1876)	2	3	72
*Uropoda cassidea* (Hermann, 1804)	0	3	1
*Uropoda splendida* (Kramer, 1882)	2	0	1
*Veigaia cervus* (Kramer, 1876)	0	8	62
*Veigaia kochi* (Trägarth, 1901)	0	0	11
*Veigaia mollis* Karg, 1971	0	0	11
*Veigaia nemorensis* (C. L. Koch, 1839)	17	38	1335
*Veigaia planicola* (Berlese, 1892)	0	0	1
*Vulgarogamasus kraepelini* (Berlese, 1904)	0	0	19
*Zercon gurensis* Mihelcic, 1962	20	19	259
*Zercon triangularis* C. L. Koch, 1836	2	1	18
Total	89	211	4549

n—number of samples collected

Our study revealed statistically significant (KW: χ^2^ = 8.406, *df* = 2, *p* = 0.015) differences in species richness per sample between broadleaved and both mixed and coniferous forests. Species richness decreased from mixed forest, through coniferous to broadleaved forest. Density (abundance m^−2^) differed significantly between mixed and broadleaved forests, however both did not differ from coniferous forest (KW: χ^2^ = 6.313, *df* = 2, *p* = 0.043). The highest values were recorded from mixed forests and the lowest from broadleaved forests. Species diversity (*H*′) did not differ statistically significantly among the three forest types, however it was close to significance (KW: χ^2^ = 5.672, *df* = 2, *p* = 0.059; Table 3).

**Table 3:** Comparison of density (abundance/m^2^), species richness and diversity among forest types assessed using Kruskal–Wallis tests

Parameter	Forest type			*df*	χ^2^	*P*
	Coniferous	Broadleaved	Mixed			
Species richness per sample	4.91 ± 0.17^a^	2.88 ± 1.57^b^	6.90 ± 1.35^a^	2	8.406	0.015
Species diversity per sample (H′)	1.18 ± 0.35^a^	0.70 ± 0.36^a^	1.59 ± 0.22^a^	2	5.672	0.059
Density (no. individuals m^−2^)	3510 ± 1169^ab^	2781 ± 2056^b^	5275 ± 1250^a^	2	6.313	0.043

For each forest types we provided mean ± SE calculated from mean values per plot. Groups marked by the same letter within rows did not differ significantly (*p* > 0.05) according to Fisher’s *posteriori* test

### Environmental factors

**Fig. 2:**
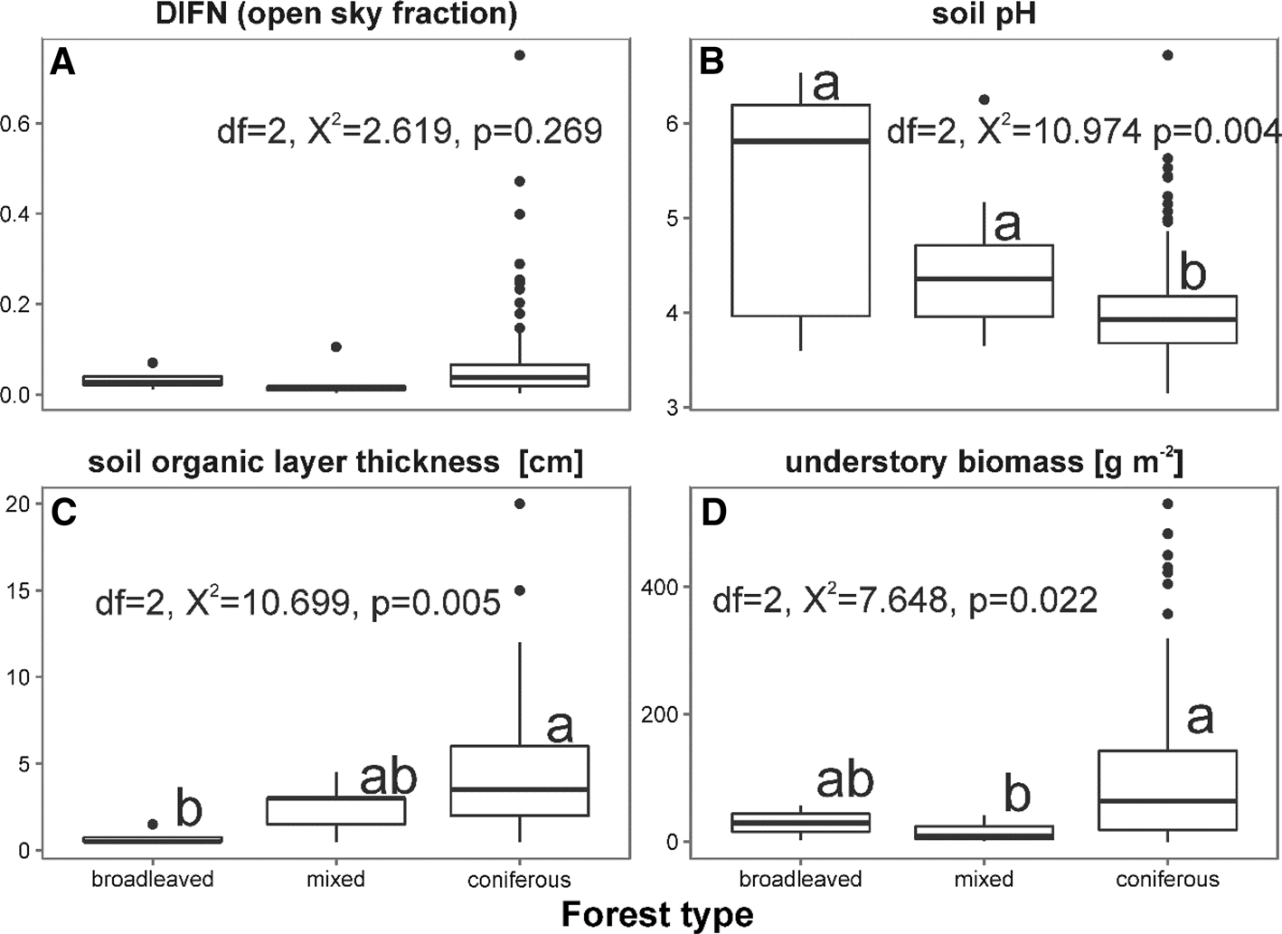
Boxplots showing differences in light conditions (**a**), soil pH (**b**), soil organic layer thickness (**c**) and understory biomass (**d**) among the forest types along the Czerwona Woda River. Top and bottom of each box represents upper and lower quartiles, respectively, a thick line inside the box indicates the median, whiskers represent range of minimum and maximum values (without outlier observations, marked by dots). *P* values refer to Kruskal–Wallis tests of differences among forests types; groups marked by the same letter above bars did not differ significantly (Fisher’s *posteriori* test: *p* > 0.05)

Overall, we found statistically significant differences between the forest types in soil pH, soil organic layer thickness and understory biomass (Fig. 2). Soil pH was lowest in coniferous forest and differed from both mixed and broadleaved stands (KW: χ^2^ = 10.974, *df* = 2, *p* = 0.004). Moreover, broadleaved forests had the lowest soil organic layer thickness, which differed from coniferous but not from mixed forests (KW: χ^2^ = 10.699, *df * = 2, *p* = 0.005). Additionally, understory biomass differed between mixed and coniferous stands (KW: χ^2^ = 7.648, *df* = 2, *p* = 0.022). The sampled forests did not differ in light conditions (KW: χ^2^ = 2.619, *df * = 2, *p* = 0.27).

### Impact of environmental factors on mite communities

**Fig. 3:**
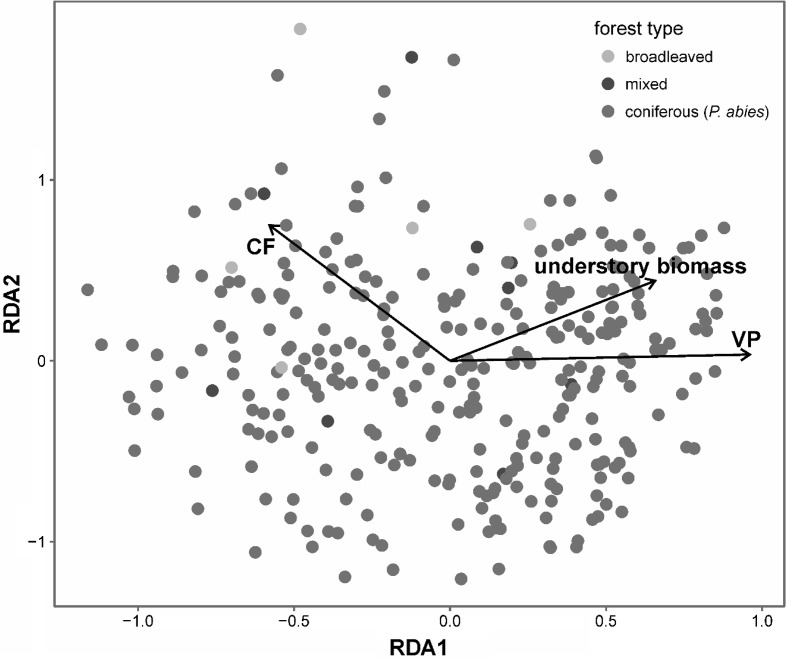
Redundancy analysis (RDA) conducted on Mesostigmata mite communities (n = 323) with environmental variables fitted in the final RDA model. Explanations of parameters and goodness of fit are shown in Table 4

RDA of mite communities revealed lack of forest type clustering within ordination space—points representing different tree stand types were scattered throughout the whole ordination space. Constrained components explained 3.6% of variance in mite species composition while unconstrained axes RDA1 and RDA2 explained 9.0 and 6.7% of the variance, respectively (Fig. 3, Table 4). In the final model, constrained components reflected understory plant parameters: biomass and proportional abundance of the plants representing *Carpino*-*Fagetea sylvaticae* and *Alno glutinosae*–*Populetea albae* classes. However, correlations of these factors were weak, but statistically significant (Table 4). This model AIC was − 135.99 whereas the AIC of the model with all parameters hypothesized was − 134.60 and AIC of the null model (intercept only) was − 130.06. Thus, forest type was not included in the final model.

**Table 4:** Fit of environmental traits to redundancy analysis (RDA) results evaluated using a PERMANOVA test (999 iterations) performed using the *vegan::anova.cca()* function (Oksanen et al. 2018)

Parameter	Abbreviation	*df*	Variance	F	Pr (> F)
Understory plants biomass	Understory biomass	1	0.00905	4.4934	< 0.001
Cover proportion of *Carpino*-*Fagetea sylvaticae* and *Alno glutinosae*–*Populetea albae* classes plants in understory	CF	1	0.00737	3.6581	< 0.001
Cover proportion of *Vaccinio*-*Piceetea* class plants in understory	VP	1	0.00776	3.8531	< 0.001
Residuals	–	319	0.64230	–	–

## Discussion

### Environmental factors and abundance, species richness and diversity

We found significant differences in species richness per sample between broadleaved and both mixed and coniferous forests, but not between mixed and coniferous. The differences in species richness between the studied forests were expected as the tree species differ in their impacts on soil parameters such as soil pH, C/N ratio and microbial activity (Priha et al. 2001; Mueller et al. 2012). Additionally, species diversity (*H*′) per sample did not differ among the forests studied (Table 3). However, we have recorded the lowest species richness per sample (2.88) in broadleaved forests, which is in contrast to other studies (Huhta and Niemi 2003). This unexpected result is probably a reflection of uneven sampling effort (coniferous: N = 162 plots; mixed: N = 5; broadleaved: N = 4); however, the differences reflected the proportion of the forest types along the Czerwona Woda River. It should also be noted that broadleaved and mixed plots exist as small patches within the surrounding monocultures of Norway spruce. On the other hand, analysis of the species accumulation curves, especially when low numbers of samples were analyzed, showed interesting tendencies. Generally, the curve for broadleaved forests started to increase rapidly and reached higher values than for either mixed or coniferous forest (Fig. 1b). Therefore, we could expect higher species richness in broadleaved forests with increasing sampling effort. This assumption could be supported by a complex survey of 10 invertebrate taxonomic groups conducted in a forest experiment covering 14 tree species that revealed that species richness of four of the groups differed statistically significantly between evergreen and deciduous tree stands (Mueller et al. 2016). These authors found higher species richness of Nematoda, Carabidae and Staphylinidae and lower richness of Oribatida in deciduous tree stands. Mesostigmata reached higher, but statistically insignificant species richness in coniferous tree stands.

Our study revealed differences in mite density (abundance m^−2^) between broadleaved and mixed forest which, but neither differed from coniferous forest. This result was similar to Migge et al. (1998), who indicated no difference in the density of Oribatida between mixed (*Picea* and *Fagus*) and spruce forests. On the other hand, Huhta and Niemi (2003) found that total abundance of Oribatida and Mesostigmata was lower in birch forest on former spruce sites when compared to natural coniferous forest. They also reported differences among sampled natural deciduous forests, therefore their results were ambiguous.

We expected differences in Mesostigmata species composition between spruce and broadleaved or mixed forests, as they differ in their impacts on soil parameters such as soil pH, C/N ratio and microbial activity (Priha et al. 2001; Mueller et al. 2012). Among four parameters studied, our study revealed differences between the forests in soil pH, soil organic layer thickness and understory biomass, but not light availability. Additionally, all three measured parameters differed statistically and revealed different trends (Fig. 2). For example, soil pH was lowest in coniferous monocultures and it differed from both mixed and broadleaved forests, whereas soil organic layer thickness was lowest in broadleaved forests and differed from coniferous forests. In reference to soil acidity, our studies were similar to previous records. Lower pH was also found in pure spruce forests when compared to mixed (spruce and birch) stands (Brandtberg et al. 2000). Therefore the question arises: How can soil pH affect Mesostigmata mite communities? Soil pH impacts soil bacteria and fungi (Mulder et al. 2005; Fierer et al. 2009), which play roles as food resources for Mesostigmata prey and secondary decomposers, i.e. collembolas (Koehler 1991) or nematodes that graze in the soil (Xiao et al. 2014). Moreover, it has been documented that some mite species feed on certain animal groups, for example *V. nemorensis* is a predator of Collembola (Koehler 1991). On the other hand, Huhta and Niemi (2003) stated that Mesostigmata as unspecialized predators, are less dependent on acidity and thickness of litter, and therefore their communities showed less specialization to forest stand type. Although, these mites seem to be less dependent to environmental factors, we found the lowest thickness of soil organic layer depth in broadleaved forest, where mite abundance, species richness and diversity (*H*′) were the lowest (Table 3). This factor is a proxy for leaf litter parameters. In general, broadleaved litter decomposes faster than needle litter, at least initially (Taylor and Wolters 2005; Horodecki and Jagodziński 2017). Moreover, Mueller et al. (2016) found that litter C/N ratio (a driver of the litter accumulation process), was the most important predictor of Mesostigmata species richness. Low importance of other parameters, especially light availability, which is claimed to drive soil temperature (by canopy transmittance; von Arx et al. 2012), was unexpected. The studies of Manu et al. (2016) indicated that temperature was a significant factor for some Mesostigmata such as *Protogamasellus pygmaeus* or *Trachytes irenae*. This could indicate that the major factor that influenced species richness near the Czerwona Woda River was soil organic layer depth, which is connected with soil pH and primarily to the tree species. Our study also indicated that forests along the Czerwona Woda River were similar. RDA analysis pointed out that environmental factors did not group with the habitat types, thus broadleaved (red points) and mixed (green) forests were randomly located on the plot among the Norway spruce matrix (Fig. 3). On the other hand, both axes explained over 15% of the variance in species composition, which indicated low variability of species composition.

### Recovery potential and diversity ‘hotspots’

Overall, we have recorded 57 species and all of them occurred in Norway spruce forests. No unique species were found in forest types sampled along the Czerwona Woda River. Although we could only speculate on the total species richness in mixed and broadleaved forests, our study revealed the complete list of Mesostigmata for spruce monocultures planted 100 years ago in place of the previous forests. This is supported by Fig. 1a, because the species accumulation curve reached an asymptote. Importantly, on the basis of collected data, we found that after 100 years the spruce monocultures changed the environment, and thus created a certain mite community and finally changed the energy channel from bacterial to fungal. This is supported by the highest soil organic layer thickness which accumulates in the forest floor and the lowest pH recorded, from coniferous plots in our study. As was stated by Zieger et al. (2015) the bacterial energy channel is assumed to be important in leaf litter material of high quality, which is decomposing quickly, such as in mixed and broadleaved forests in our study. Therefore, we considered the next question: How large is the recovery potential of mite communities in those forests? Generally, it seems to be low as the valley of the Czerwona Woda River contains only small areas of mixed and broadleaved forests (nine study plots reflected their abundance; Pielech et al. 2018), which can play the role of ‘hotspots’ of mite species diversity. Unfortunately, our study revealed low proportional abundance of mites from the suborder Uropodina along the Czerwona Woda River, which was similar in the three types of forests and varied from ca. 10–15%. Generally, higher proportional abundance of those mites is considered as characteristic for deciduous forests (Koehler 1991), where they, as mites with low mobility, feed mainly on nematodes consuming bacteria. Moreover, Uropodina were represented by only nine species in our study (Table 2), which could be considered as very low in comparison to the results of Napierała et al. (2009), who noted 28 species in soil environments of the oak-hornbeam forest. Generally, our results were similar to studies conducted in artificially introduced spruce forests. For instance, Skorupski et al. (2008) recorded six Uropodina species in spruce forests damaged by industrial pollution in the Izera Mountains. Surprisingly, all of the Uropodina species recorded by them were reported from spruce forests in our studies, and the reported proportional abundance of the Uropodina was similar in their study and reached ca. 13%. On the other hand Skorupski et al. (2009) reported lower total species richness (23 species) including five species of Uropodina from pure mature spruce forests in the Karkonosze Mountains. Therefore, low proportional abundance and species richness of Uropodina seems to be typical for artificially introduced spruce forests in mountain areas, including the vicinity of mountain streams.

Surprisingly, we have recorded in total 30 species (52.6% of all species) from only nine mixed and broadleaved plots (5.2% of the sampled plots) which indicated high recovery potential of the undisturbed plots. Moreover, those forests seem to be very important for rare species occurring in low densities such as *Pachylaelaps bellicosus*, *Trachytes montana* or *Veigaia mollis* which clearly responded positively to forest conversion from pure spruce to mixed forests (Skorupski et al. 2009). We have also recorded other species typical for broadleaved mountain forests such as *Macrocheles* sp., *Olodiscus minima*, *O*. *misella*, *Pachylaelaps longisetis*, *Polyaspinus cylindricus*, *Uropoda cassidea* and *U. splendida* (Huhta and Räty 2005; Sabbatini Peverieri et al. 2008; Diaz-Aguilar et al. 2013; Napierała et al. 2015) which still occur in spruce forests in our study. Therefore we could expect changes in mite communities as a reaction to ongoing forest conversion. Our results suggest that the vicinity of the Czerwona Woda River is still inhabited by rare species or species restricted in distribution to specific microhabitats, although these species occur in low densities.

## Conclusions

Our study indicated that introduction of spruce monocultures in place of natural mixed and broadleaved forests along the mountain river changed soil pH, soil organic layer thickness and understory biomass. Although coniferous forests did not differ from either broadleaved or mixed forests in reference to mite density (individuals × m^−2^) and species diversity (H′), they were characterized by low species richness and abundance of Uropodina—a characteristic component of the broadleaved forests. Additionally, although the mite communities in spruce monocultures were inhabited by common mite species, there is still high recovery potential, as the communities still maintain rare species typical for broadleaved and mixed forests.
